# Supplementary material for: The genomic basis of copper tolerance in Drosophila is shaped by a complex interplay of regulatory and environmental factors
Source: BMC Biol. 2022 Dec 8;20:275. doi: 10.1186/s12915-022-01479-w (PMC9733279; doi:10.1186/s12915-022-01479-w)

**Figure S1. Copper tolerance phenotypes across all populations.**
**A)** $LT_{50}$ values for the 71 natural strains collected in 2015 by the DrosEU consortium (see Additional File 4: Table S2A for the name of each strain). The majority of these strains were inbred between 15-20 generations before screening (Additional File 2: Table S1A). **B)** $LT_{50}$ values for the 26 isofemale strains from additional locations in Austria, Portugal and Italy collected in 2011 and 2018. The strains in each chart are arranged in order of ascending $LT_{50}$ (see Additional File 4: Table S2A). Bar colour corresponds to copper concentration, as per the map in Additional File 1: Fig. 1A. The cooler colours refer to low copper concentrations and the warmer colours refer to high copper concentrations. Error bars represent 95% CI of the probit slope. The pink labels in **A)** represent the three sensitive strains and the blue labels represent the three tolerant strains that were subject to subsequent differential expression analysis and genome sequencing.

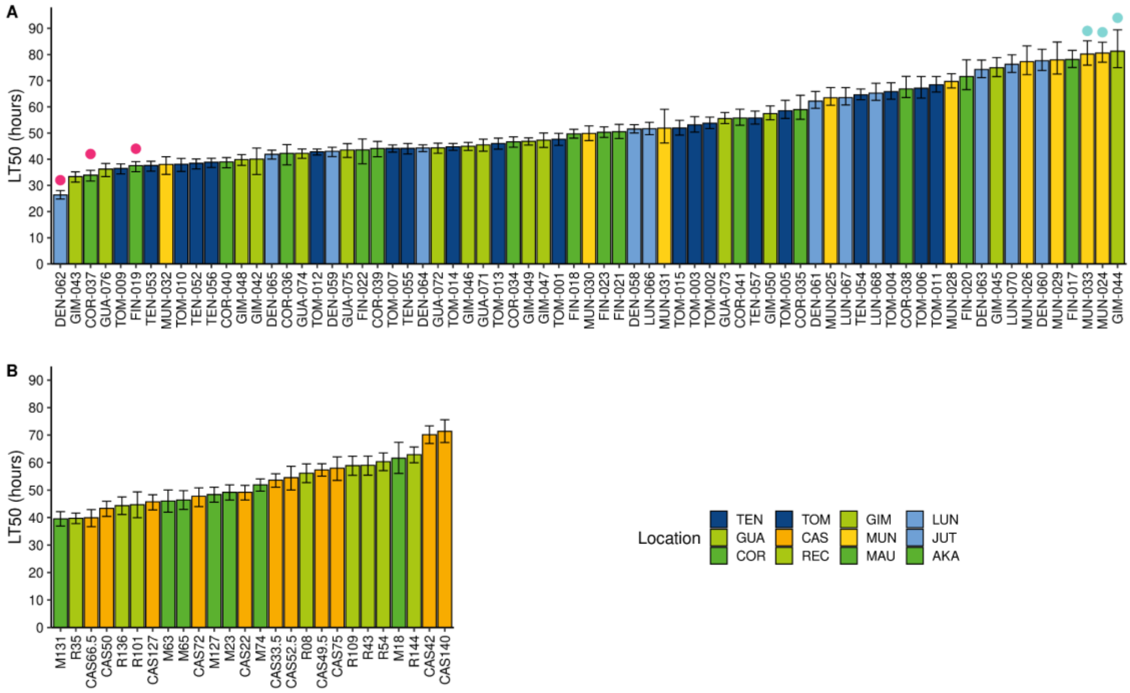

Supplement: Supplementary file 2 — Additional file 2: Figure S1. Copper tolerance phenotypes across all populations. [file 12915_2022_1479_MOESM2_ESM.pdf]
