# Supplementary material for: The genomic basis of copper tolerance in Drosophila is shaped by a complex interplay of regulatory and environmental factors
Source: BMC Biol. 2022 Dec 8;20:275. doi: 10.1186/s12915-022-01479-w (PMC9733279; doi:10.1186/s12915-022-01479-w)

Figure S2. DGET expression analysis for gut subsections.
Breakdown of gene expression levels for tolerant (top) and sensitive (bottom) strains. Subsections: a = Anterior (regions 1-3); Cu = Copper Cells; Fe = Iron Cells; LFCs = Large Flat Cells; p = Posterior (regions 1-4). With the exception of the a1 segment of the gut in tolerant strains, genes found highly and extremely highly expressed in all sub-sections were significantly enriched for our DEGs.

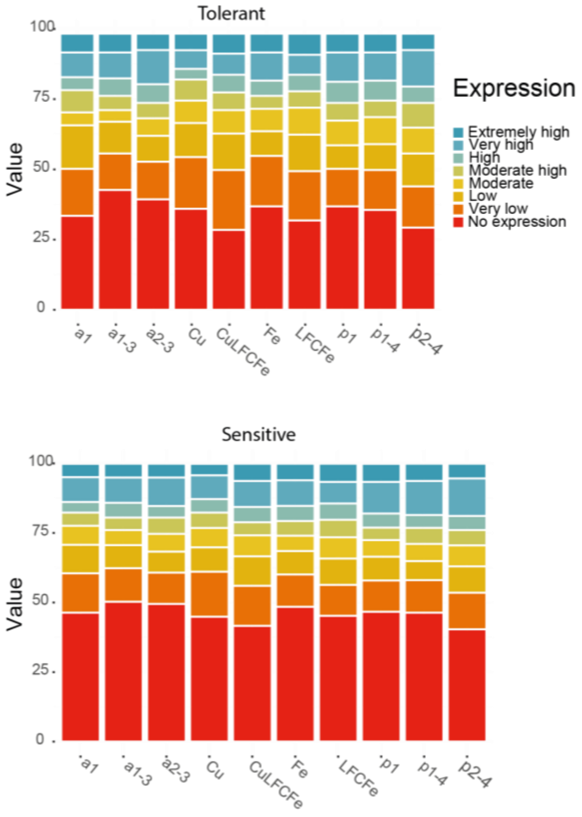

Supplement: Supplementary file 6 — Additional file 6: Figure S2. DGET expression analysis for gut subsections. [file 12915_2022_1479_MOESM6_ESM.pdf]
