# Supplementary material for: The genomic basis of copper tolerance in Drosophila is shaped by a complex interplay of regulatory and environmental factors
Source: BMC Biol. 2022 Dec 8;20:275. doi: 10.1186/s12915-022-01479-w (PMC9733279; doi:10.1186/s12915-022-01479-w)

Figure S3. GO clustering analysis for DEGs between tolerant and sensitive strains in basal conditions.

Top enriched GO terms associated with the DEGs when comparing tolerant *vs.* sensitive strains under control conditions. The *y*-axis indicates gene functions, and the *x*-axis indicates the proportion of total DEGs in a given GO category (gene ratio).

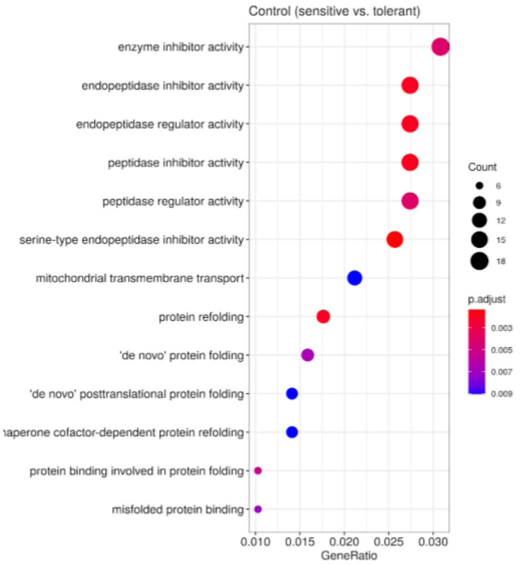

Figure S4. MMC analysis of DEGs before and after copper exposure.
Analysis was performed separately for both tolerant (**top**) and sensitive (**bottom**) strains. Treated samples are shown on the left (**A, C**) and control on the right (**B, D**). Each coloured point represents the Spearman correlation (*r*s) between two genes. Across the tolerant strains, we identified 24 modules with an average positive correlation, |r|, of 0.72 from treated samples, and 17 modules with a |r|=0.65 from controls, indicating a higher level of expression co-ordination after copper exposure. For sensitive strains, 21 modules were identified in the treated samples, with an average |r|=0.77; and 40 in the controls with and |r|=0.71, with a less pronounced degree of partitioning, indicating that expression in sensitive strains is actually less modulated after 24 hours of copper exposure (see also Additional File 7: Table S8).

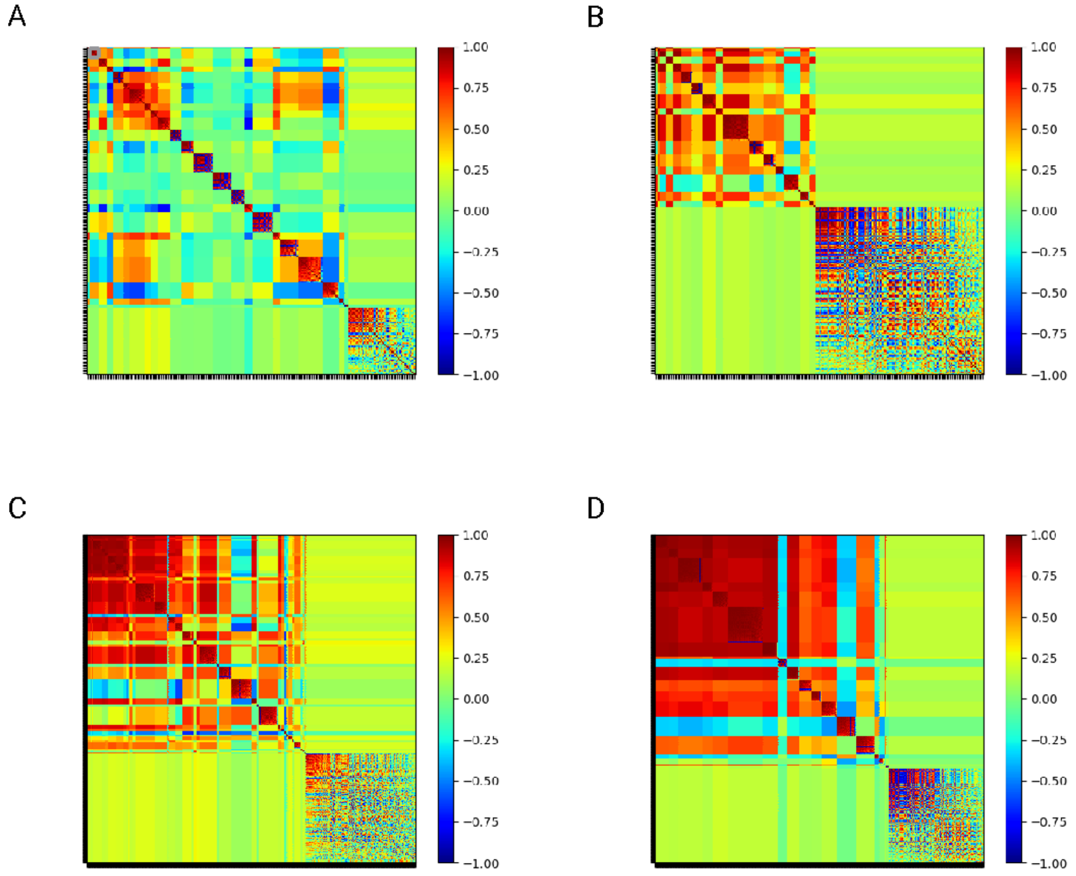

Figure S5. Overlapping expression patterns between copper DEGs and regulatory factors knockout and knockdowns.

A) Overlap between DEGs after copper exposure and genes regulated by *sir2* and *HNF4*. Venn diagrams showing the degree of overlap between the DEGs between *Sir2* and *HNF4* knockouts along with tolerant (left) and sensitive strains (right). **B)** Venn diagrams showing the degree of overlap between the DEGs for two additional downstream targets of *sir2*. Overlap between DEGs from tolerant strains and *DHR96* knock-outs (top left), sensitive strains and *DHR96* knock-outs (top right), tolerant strains and *dFoxo* knock-outs (bottom left) and sensitive strains and *dFoxo* knock-outs (bottom right). The numbers represented in red are found commonly up-regulated, those in blue commonly down-regulated and those in yellow are discordant.

A

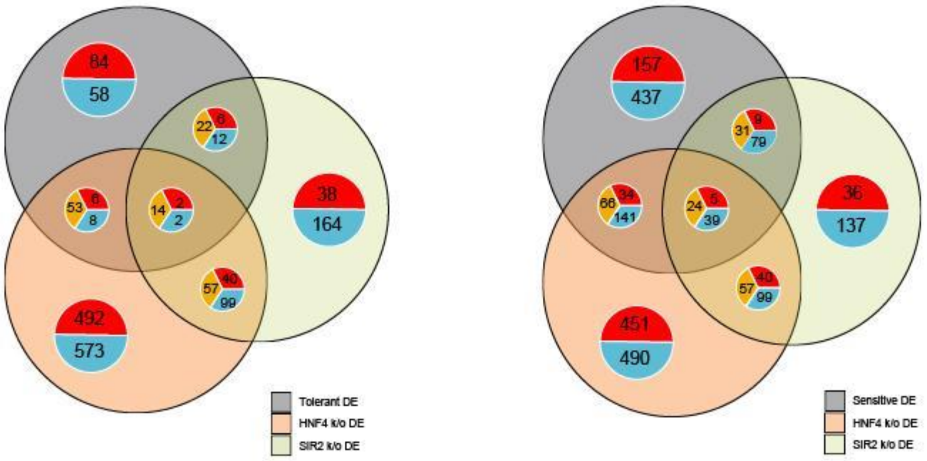

B

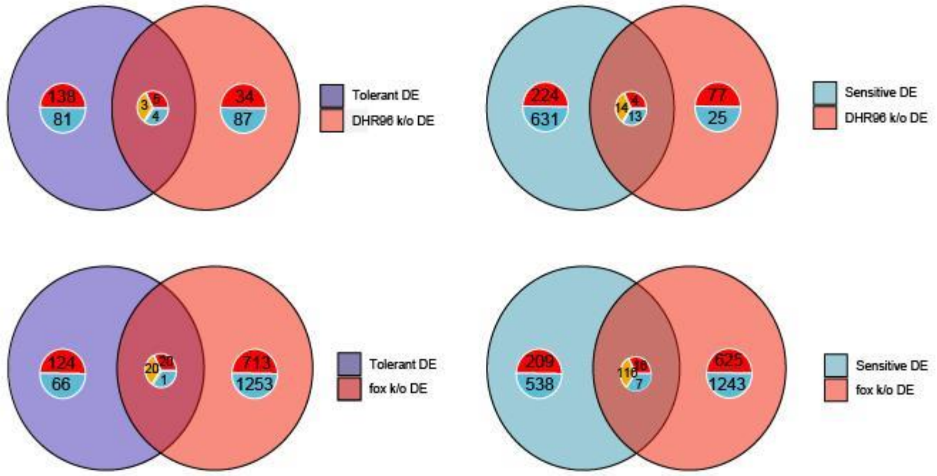

Supplement: Supplementary file 8 — Additional file 8: Figures S3. GO clustering analysis. Figure S4. Modulated Modularity Cluster analysis. Figures S5. Overlapping between DEGs and regulatory factors. [file 12915_2022_1479_MOESM8_ESM.pdf]
