# Supplementary material for: The genomic basis of copper tolerance in Drosophila is shaped by a complex interplay of regulatory and environmental factors
Source: BMC Biol. 2022 Dec 8;20:275. doi: 10.1186/s12915-022-01479-w (PMC9733279; doi:10.1186/s12915-022-01479-w)

**Figure S6. Kaplan-Meier survival curves for the survival assays performed on RNAi knockdowns and disruption mutants for all gene candidates.**
Shaded regions indicate the 95% confidence intervals. Statistical significance was estimated by using log-rank tests. Plots shaded in blue are the knockdown or disruption lines and plots in red are the control lines.

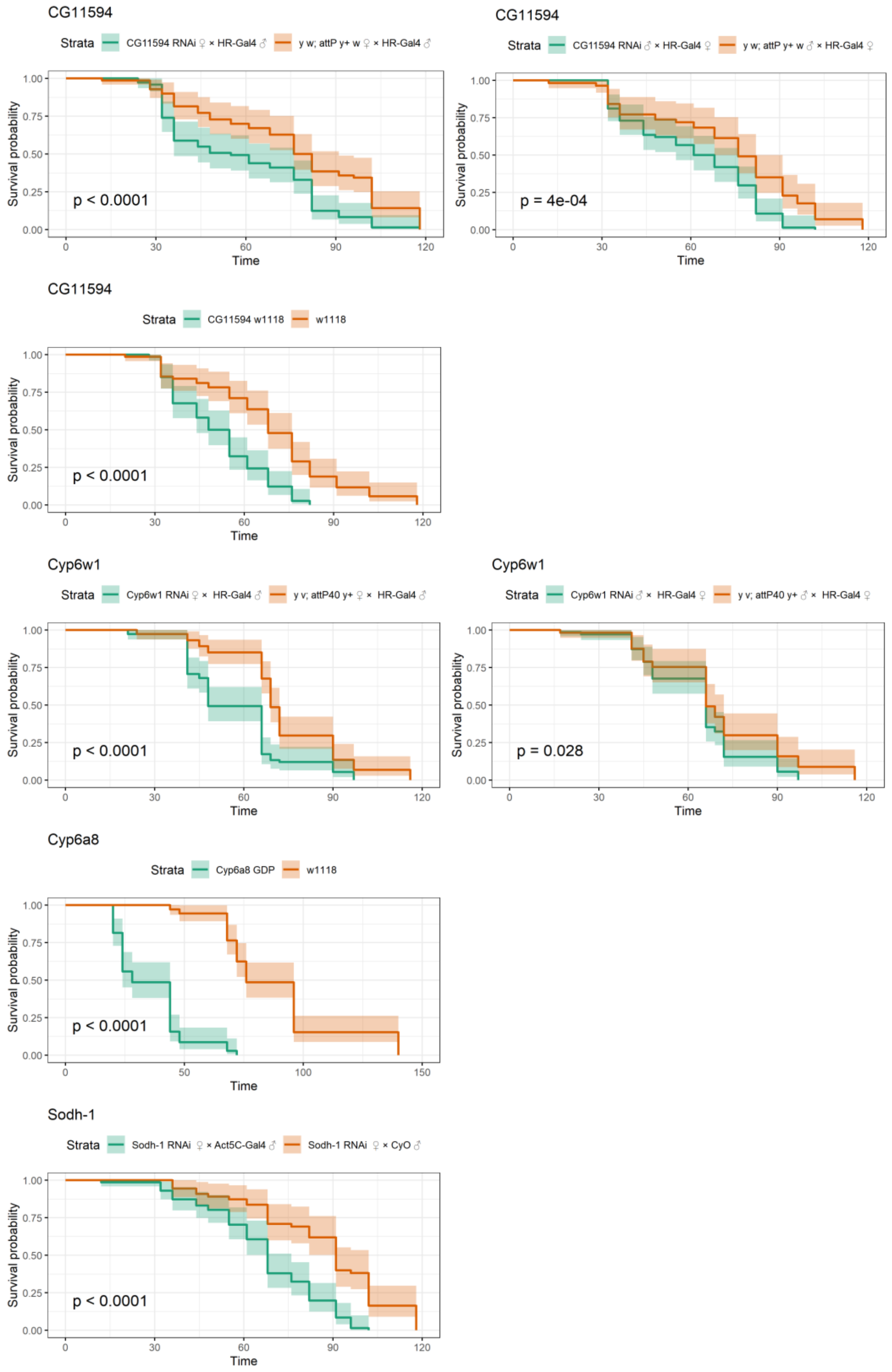

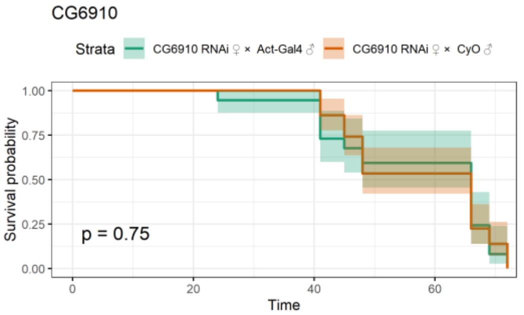
CG6910
Strata
CG6910 RNAi ♀ × Act-Gal4 ♂
CG6910 RNAi ♀ × CyO ♂
Survival probability
p = 0.75
Time

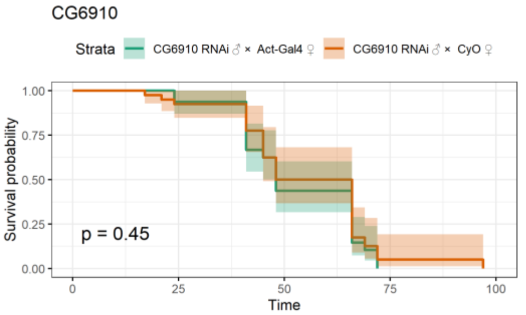
CG6910
Strata
CG6910 RNAi ♂ × Act-Gal4 ♀
CG6910 RNAi ♂ × CyO ♀
Survival probability
p = 0.45
Time

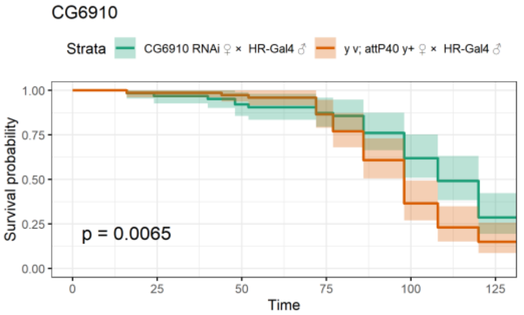
CG6910
Strata
CG6910 RNAi ♀ × HR-Gal4 ♂
y v; attP40 y+ ♀ × HR-Gal4 ♂
Survival probability
p = 0.0065
Time

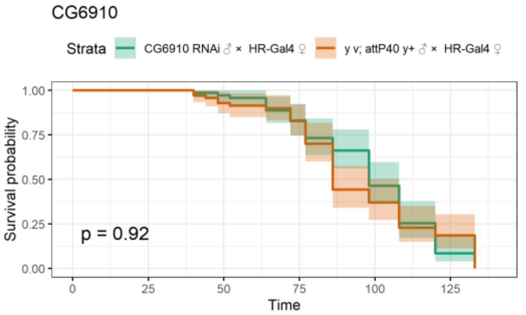
CG6910
Strata
CG6910 RNAi ♂ × HR-Gal4 ♀
y v; attP40 y+ ♂ × HR-Gal4 ♀
Survival probability
p = 0.92
Time

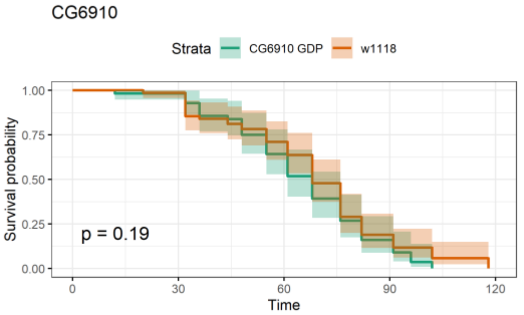
CG6910
Strata
CG6910 GDP
w1118
Survival probability
p = 0.19
Time

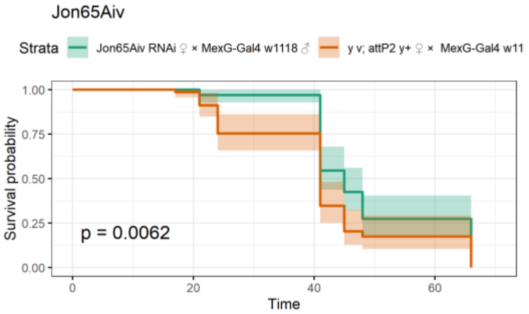
Jon65Aiv
Strata
Jon65Aiv RNAi ♀ × MexG-Gal4 w1118 ♂
y v; attP2 y+ ♀ × MexG-Gal4 w11
Survival probability
p = 0.0062
Time

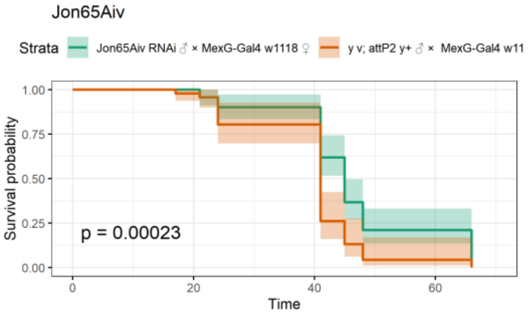
Jon65Aiv
Strata
Jon65Aiv RNAi ♂ × MexG-Gal4 w1118 ♀
y v; attP2 y+ ♂ × MexG-Gal4 w11
Survival probability
p = 0.00023
Time

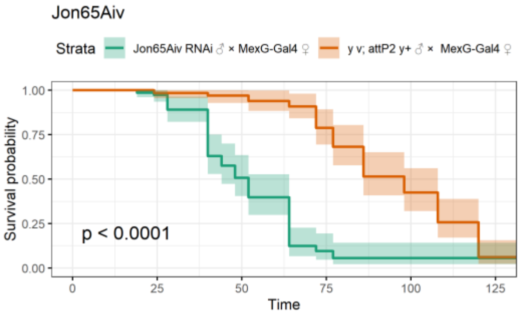
Jon65Aiv
Strata
Jon65Aiv RNAi ♂ × MexG-Gal4 ♀
y v; attP2 y+ ♂ × MexG-Gal4 ♀
Survival probability
p < 0.0001
Time

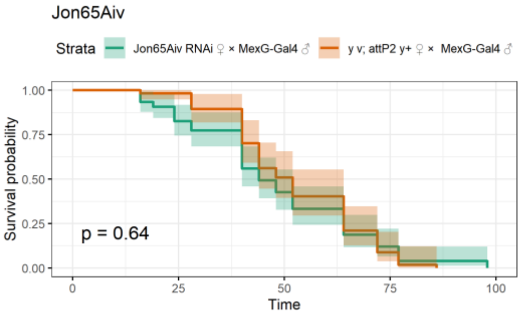
Jon65Aiv
Strata
Jon65Aiv RNAi ♀ × MexG-Gal4 ♂
y v; attP2 y+ ♀ × MexG-Gal4 ♂
Survival probability
p = 0.64
Time

CG32444

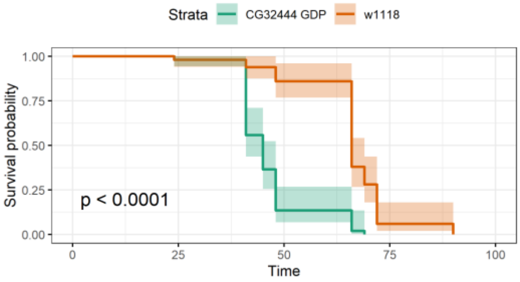


CG5966

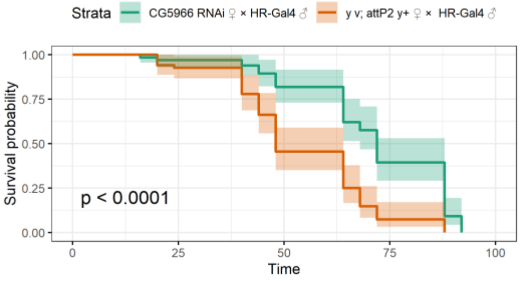


CG5966

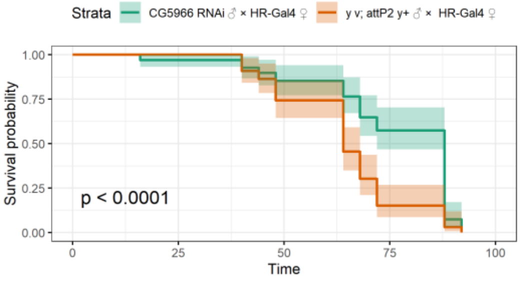


CG5773

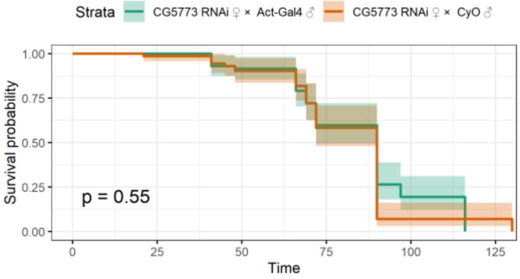


Cyp4e3

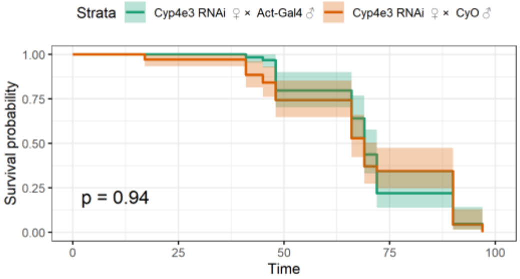


Cyp4e3

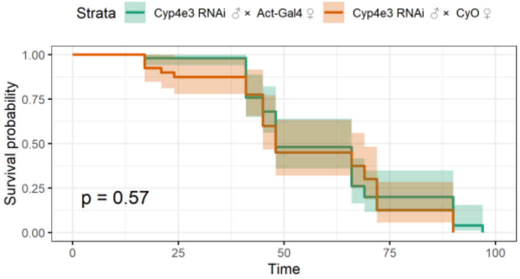


Cyp4e3

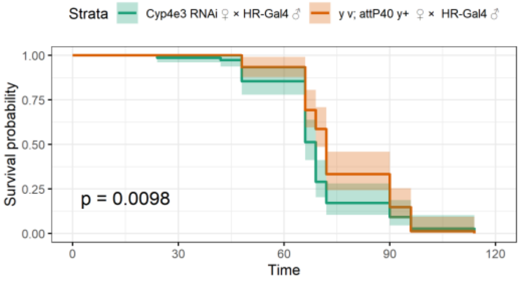


Cyp4e3

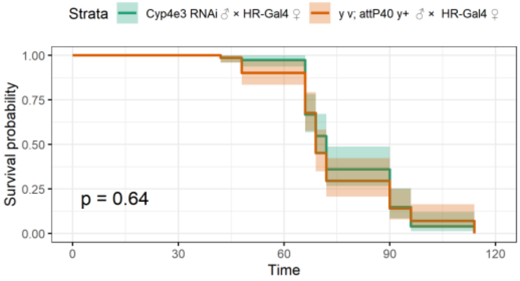

Supplement: Supplementary file 9 — Additional file 9: Figure S6. Kaplan-Meier survival curves for the survival assays performed on RNAi knockdowns and disruption mutants for all gene candidates. [file 12915_2022_1479_MOESM9_ESM.pdf]
