# Supplementary material for: The genomic basis of copper tolerance in Drosophila is shaped by a complex interplay of regulatory and environmental factors
Source: BMC Biol. 2022 Dec 8;20:275. doi: 10.1186/s12915-022-01479-w (PMC9733279; doi:10.1186/s12915-022-01479-w)

Figure S7. Kaplan-Meier survival curves for the survival assays performed on outbred populations with and without the three candidate TE insertions.
Shaded regions indicate the 95% confidence intervals. Statistical significance was estimated by using log-rank tests. Those plots shaded in red are the outbred populations with the TE insertion and plots in blue are the outbred populations without the TE insertion.

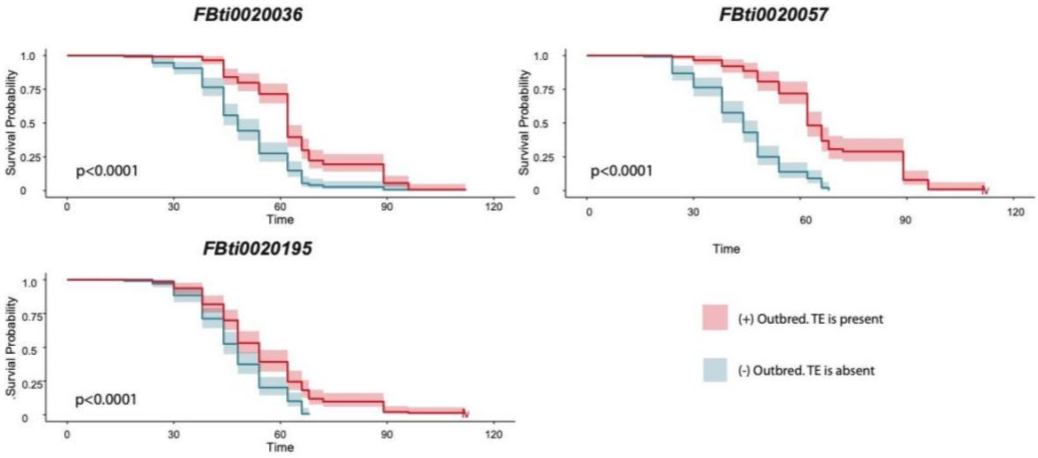

Supplement: Supplementary file 13 — Additional file 13: Figure S7. Kaplan-Meier survival curves for the survival assays performed on outbred populations with and without the three candidate TE insertions. [file 12915_2022_1479_MOESM13_ESM.pdf]
